# Detecting Site‐Level Fraud via an Artificial Intelligence/Machine Learning Paradoxical Patient Analysis in an Alzheimer's Disease Clinical Trial

**DOI:** 10.1002/alz70859_103051

**Published:** 2025-12-25

**Authors:** Joseph Geraci, Patrick P O'Keefe, Bessi Qorri, Paul Leonchyk, Larry Alphs, Luca Pani, Kent Hendrix, Samuel P. Dickson, Suzanne B. Hendrix

**Affiliations:** ^1^ NetraMark Corp, Toronto, ON Canada; ^2^ Department of Pathology and Molecular Medicine, Queen’s University, Kingston, ON Canada; ^3^ Arthur C. Clarke Center for Human Imagination, School of Physical Sciences, University of California, San Diego, La Jolla, CA USA; ^4^ Pentara Corporation, Salt Lake City, UT USA; ^5^ Department of Psychiatry and Behavioral Sciences, Leonard M. Miller School of Medicine, University of Miami, Coral Gables, FL USA

## Abstract

**Background:**

Ensuring data integrity in Alzheimer’s Disease (AD) clinical trials is crucial, yet the subjective nature of endpoints and potential site‐level inconsistencies can undermine study outcomes. Paradoxical patients, defined as individuals whose data clustering patterns deviate significantly from expected norms, may indicate anomalies such as fraudulent or unrepresentative behavior. We developed and applied NetraAI, a machine learning (ML) framework leveraging information‐theoretic methods and clustering penalties, to detect paradoxical patients and indirectly assess site‐level anomalies. Our aim was to determine whether analyzing patient‐level data alone could uncover both patient‐specific and site‐specific fraudulent activities.

**Method:**

Using data from approximately 200 AD patients enrolled in a clinical trial, NetraAI generated a Paradoxical Patient Risk Score by simulating hundreds of patient geometry scenarios using only baseline and screening variables, including clinical scale data (ADAS‐Cog, ADCS‐ADL, MMSE, CDR‐SB, NPI), medical data (vital signs, medical history, and labs), and adverse events and pharmacokinetic assessments. The score integrated information‐theoretic variability, singleton occurrence frequencies, and co‐association penalties, enabling robust anomaly detection at the patient level. Patients were ranked by their paradoxical scores and their corresponding sites were mapped post‐hoc. These results were compared to independently empirically‐derived site “true fraud scores” (based on factors outside of screening and baseline factors) withheld during initial analyses to validate our findings.

**Result:**

The NetraAI findings aligned with the holdout fraud data. Notably, Site G hosted the largest concentration of top‐ranked paradoxical patients, aligning with its true fraud score of 6.89. Conversely, Site R ranked as low‐risk by patient‐derived scores, matching its low fraud score of 2.21. Across all sites, strong correlations emerged between patient‐driven scores and true fraud scores, with top‐ranked sites such as G and M consistently flagged as problematic.

**Conclusion:**

This work demonstrates that NetraAI’s paradoxical patient‐focused approach can identify anomalous patients and sites in AD clinical trials using screening and baseline data alone, even without prior site‐level fraud data. By relying solely on patient‐level metrics, NetraAI provides a scalable, objective strategy to enhance data integrity in clinical trials. Future research will extend these insights to direct site‐level assessments, further refining this framework for broader clinical trial applications.

